# Screening for the presence of *mcr-1/mcr-2* genes in Shiga toxin-producing *Escherichia coli* recovered from a major produce-production region in California

**DOI:** 10.1371/journal.pone.0187827

**Published:** 2017-11-08

**Authors:** Daniela Mavrici, Jaszemyn C. Yambao, Bertram G. Lee, Beatriz Quiñones, Xiaohua He

**Affiliations:** 1 Foodborne Toxin Detection and Prevention Research Unit, Western Regional Research Center, Agriculture Research Service, U.S. Department of Agriculture, Albany, California, United States of America; 2 Produce Safety and Microbiology Research Unit, Western Regional Research Center, Agriculture Research Service, U.S. Department of Agriculture, Albany, California, United States of America; Ross University School of Veterinary Medicine, SAINT KITTS AND NEVIS

## Abstract

The rapid spreading of polymyxin E (colistin) resistance among bacterial strains through the horizontally transmissible *mcr-1* and *mcr-2* plasmids has become a serious concern. The emergence of these genes in Shiga toxin-producing *Escherichia coli* (STEC), a group of human pathogenic bacteria was even more worrisome, urging us to investigate the prevalence of *mcr* genes among STEC isolates. A total of 1000 STEC isolates, recovered from livestock, wildlife, produce and other environmental sources in a major production region for leafy vegetables in California during 2006–2014, were screened by PCR for the presence of plasmid-borne *mcr-1* and *mcr-2*. All isolates tested yielded negative results, indicating if any, the occurrence rate of *mcr-1*/*mcr-2* among STEC was very low in this agricultural region. This study provides valuable information such as sample size needed and methodologies for future surveillance programs of antimicrobial resistance.

## Introduction

Polymyxin E (colistin) is the antibiotic of last resort for Gram negative multidrug resistant superbugs [1]. It acts by binding the lipid A component of lipopolysaccharides and subsequently disrupting the bacterial membrane. The colistin resistant gene product, MCR, is a phosphoethanolamine transferase that catalyzes the addition of phosphoethanolamine to lipid A to decrease colistin’s binding affinity to the lipid A component of bacterial membrane [2–4], thus reducing colistin’s antibiotic activity. The use of colistin is limited to treating severe infections by gram-negative bacteria due to its toxicity in humans [5, 6], but it has been used for decades in veterinary medicine around the world to treat animal intestinal infections [7]. In 2016. Liu et al. [8] reported the first case of plasmid mediated colistin resistance *mcr-1* gene, harbored on a horizontally transmissible plasmid [9]. Since then, the plasmid mediated colistin resistance has been reported from different areas around the world in patients, livestock, pet foods and wild animals [10, 11]. In the United States, MCR-1 mediated colistin resistant strains have been isolated from patients in six states [12–17] and the resistance mechanism have even spread to extended-spectrum β-lactamase (ESBL)-producing Shiga toxin-producing *Escherichia coli* (STEC) cultured from pigs [18]. STEC O157 and non-O157 are recognized as leading cause of foodborne outbreaks and are responsible for more than 175, 900 foodborne illnesses, 2,450 hospitalizations and 20 deaths in humans each year in the United States alone for a maximum total cost of about 1.2 billion dollars [19]. In particular, fresh leafy vegetables have been implicated in foodborne outbreaks associated with STEC infections [19–20]. It is urgent to detect, map and contain the plasmid mediated colistin resistance among STEC isolates in order to prevent its further spreading in animals, produce and environment. In this study, we investigated the prevalence of the plasmid-borne *mcr-1* and *mcr-2* among STEC by PCR analysis of 1000 isolates collected from animal, produce, and environmental sources in a major agricultural region for leafy greens in California.

## Materials and methods

### Bacterial strains

**Fig 1:**
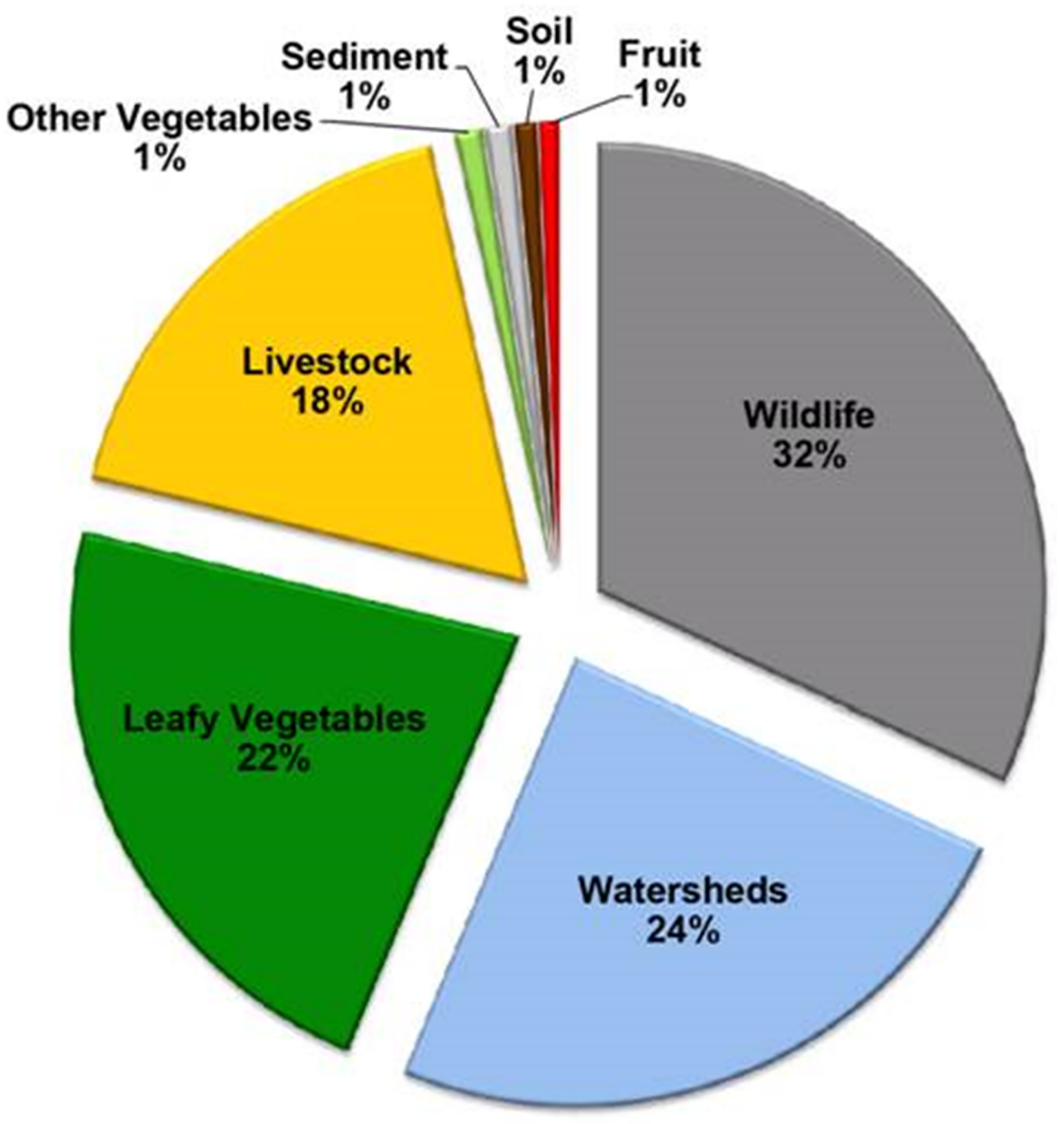
Sample sources that yielded the Shiga toxin-producing *E*. *coli* strains, examined in the present study. Wildlife (32%), watersheds (24%), leafy vegetables (22%), livestock (18%), other vegetables (1%), sediment (1%), soil (1%), and fruit (1%).

The recovery of STEC O157 and non-O157 isolates from livestock, wildlife, produce, soil, and water samples was performed by subjecting samples to a non-selective enrichment step, followed by an immunomagnetic separation, and selection of suspect STEC colonies, based on colony colors and morphologies displayed on chromogenic selective solid agar, as described in previous reports [20–22]. A subset of 1000 STEC isolates recovered from wildlife (32%), watersheds (24%), leafy vegetables (22%), livestock (18%), sediment (1%), soil (1%), fruit (1%), and other vegetables (1%) were selected for the present study (Fig 1). The examined STEC isolates were recovered from leafy greens (lettuce/spinach), soil, water, sediment, and wild animal feces collected from private produce farms and ranches in Monterey, San Benito, San Luis Obispo Counties in the central California coast, as described in previous studies [20–22]. Sampling locations sites in private produce farms and ranches were not disclosed for reasons of confidentiality [21], and voluntary permission was obtained from owners of produce farm and farmers for collection of samples [21]. Permission from private land owners was obtained for enabling USDA Wildlife Services or California Department of Fish and Game to conduct wildlife sample collection, as previously documented [21]. Additional STEC isolates from watershed samples, collected from public access sites in Monterrey County in California [22], were also examined. Given that all of the watershed sampling sites were on public lands, there were no specific permissions required for sampling [22]. These watershed sites with public access were selected in collaboration with the Central Coast Water Quality Control Board, as previously documented in a recent report [22]. The wildlife fecal sources for STEC isolates examined in the present study were collected from blackbird, cow bird, crow, Canadian geese, coyote, deer, deer mouse, elk, ground squirrel, kangaroo rat, meadow vole, feral pig, rabbit, skunk and snakes. The livestock fecal sources were collected from alpaca, cattle, dog, and goats, as documented in previous reports [20–21]. A small subset of STEC isolates from cantaloupe were provided by the Microbiological Data Program, which was previously managed by the U.S. Department of Agriculture-Agricultural Marketing Service. Bacterial cultures were propagated on Luria-Bertani (LB) agar (Difco, Detroit, MI) and stored in Microbank Vials with Cryo preservative (Pro-Lab Diagnostics, Round Rock, TX) at -80°C until further use.

As positive controls for plasmid encoded *mcr-1*, *E*. *coli* strains AR-Bank #0346 and AR-Bank #0349 were used [23]. These positive-control strains, harboring *mcr-1* on a multicopy plasmid [23], were kindly provided by FDA-CDC Antimicrobial Resistance Bank, Atlanta, GA.

### Conventional PCR

Single colonies from STEC isolates were grown aerobically in 1mL LB broth (Difco) in Corning 96-Well Assay Blocks (Corning Life Sciences, Tweksbury, MA) for 24 hr with constant shaking (200 rpm) at 37°C. Cell lysates were prepared from 100 μl of the bacterial overnight cultures, which were collected by centrifugation at 2000 ×g for 5 min. Cell pellets were resuspended in 100 μl of HyClone molecular biology-grade water (GE Healthcare Bio-Sciences, Pittsburg, PA), heated at 95°C for 20 min, and centrifuged at 2000 ×g for 5 min, and the supernatants were collected and frozen until further use. To screen for the presence of *mcr*-*1* and *mcr*-*2* in the STEC isolates, a conventional PCR assay was performed by using primers CLR5-F and CLR5-R targeting *mcr-1* [8] and primers MCR2-F and MCR2-R targeting *mcr-2* [10]. Additionally, amplifications targeting *stx*_1_ and *stx*_2_ genes, encoding Shiga toxin, and the *gadB* gene, encoding glutamate decarboxylase, were included as positive controls to confirm the source and quality of the DNA used for PCR analysis [24]. Each PCR reaction consisted of 12.5 μl of 2× GoTaq Green Master Mix (Promega Corporation, Madison, WI), 0.5 μM of each primer, and 3 μl of the bacterial crude lysate in a total volume of 25 μl with amplification conditions, as previously described [24]. Amplified products were analyzed in 2% agarose gels containing 0.04 μl/ml GelRed Nucleic Acid Stain (Phenix Research, Candler, NC).

### Real-time PCR for *mcr-1*, *mcr-2*, and 16S rRNA genes

Cell lysates (3 μL), prepared as described above, were used as DNA template, MCR-CR-F: 5’-acggcgtattctgtgccgtgtat-3’ and MCR-CR-R 5’- gctgttcttttggtgcaaaggcattt-3’ were used as primers for PCR analysis of *mcr-1/mcr-2* genes and primers UNI338F and UNI1100R [25] were used as primers for PCR analysis of the 16S rRNA gene. Two steps real-time PCR were performed using QuantiNova SYBR Green mixture and Qiagen Rotor Gene system (Qiagen, Hilden, Germany). A typical 20 μL of PCR reaction includes 0.7 μM of each primer, 3 μL of lysate and SYBR Green mixture (1x). The PCR conditions used are: 95°C, 10 minute; 30 cycles including 95°C, 15 seconds, 60°C, 1 min; and 72°C, 30 seconds. PCR cycle threshold (Ct) value was used to determine the sample’s positive and negative intervals. When the Ct value is less than the average Ct value of the positive controls plus 3, the sample is considered as positive. When the Ct value is greater than or equal the average Ct value of the positive controls plus 3, the sample is considered as negative [26].

## Results and discussion

STEC are important food-borne pathogens that cause about 35% of all bloody diarrhea in the USA and life-threatening systemic complications, including HUS and there is no effective therapy for this illness [27]. Recent studies have found the presence of transmissible plasmid-borne colistin resistance gene, *mcr-1*, in some STEC isolates [28]. Moreover, it has also been shown that *mcr* genes have already spread to the environment prior to their detection [29]. Based on these findings, the objective of this study was to assess the prevalence of *mcr-1/mcr-2* genes among STEC recovered from multiple types of samples collected in a major produce-production area in California. A total of 1000 STEC isolates collected between 2006 through 2014 covering a wide-range of collection time and sources were tested. Fig 1 shows the proportion of sources yielding the tested STEC isolates. Since the discovery of the first plasmid-borne *mcr-1* gene in November, 2015, multiple *mcr* variants have been identified [30, 31], it is possible that new variants may continue to be identified. Therefore, it is critical to have an analytical method that is capable of detecting all *mcr* genes for a surveillance program. We analyzed more than 100 *mcr* gene sequences blasted [32] from the Genbank and identified two conserved regions: 5’-acggcgtattctgtgccgtgtat-3’ and 5’- gctgttcttttggtgcaaaggcattt-3’. These two conserved regions were named as MCR-CR-F and MCR-CR-R and used as primers in the real-time PCR analysis of *mcr* genes present in STEC isolates.

**Fig 2:**
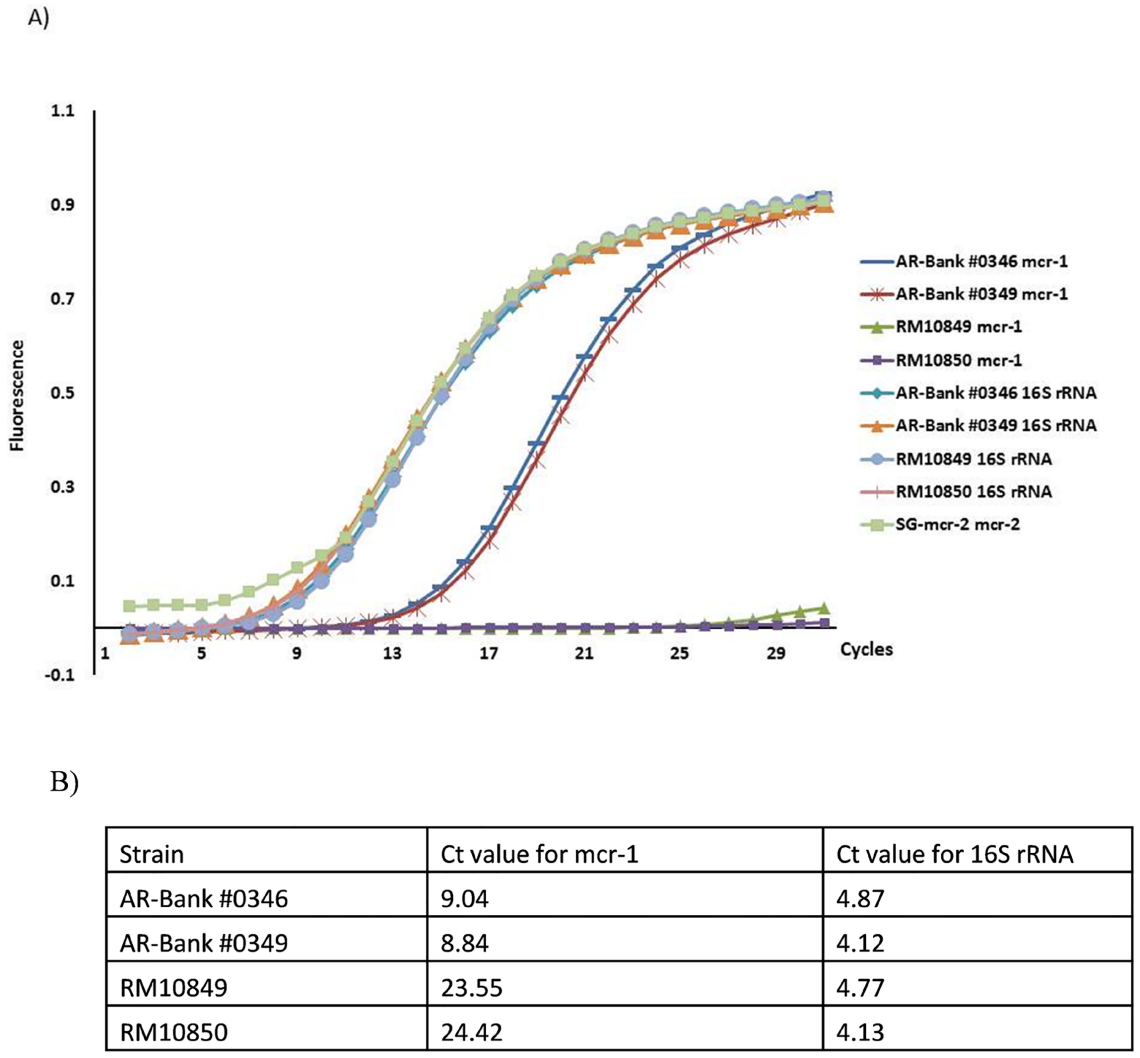
The specificity and sensitivity of real time PCR for *mcr-1/mcr-2* and 16S rRNA genes. A) Plot of the amplification of *mcr-1/mcr-2* and 16S rRNA genes to calculate their cycle threshold (Ct) values by analyzing the fluorescence curve of the PCR products. AR-Bank #0346 and AR-Bank #0349 are the *mcr-1* positive controls; MCR-2-SG is the synthetic gene of *mcr-2*, serving as the *mcr-2* positive control; RM10849 and RM10850 are the *mcr-1* negative controls. B) Ct values obtained from the real time PCR analysis.

To validate the specificity of the real-time PCR method, lysates from *mcr-1* positive strains, AR-Bank #0346 and AR-Bank #0349, and *mcr-1* negative strains, RM10849 and RM10850, were used as controls. Since *E*. *coli* strains harboring *mcr-2* gene were not available, the full *mcr-2* gene was synthesized by IDT (www.idtdna.com) based on the published sequence [10] and used as a positive control to validate the real-time PCR method for amplification of *mcr-2* gene fragment. To confirm the source and quality of the lysates used in the real-time PCR, amplification of the bacterial 16S rRNA was performed using these lysates as templates. Amplicons generated from *mcr-1* and 16S rRNA control samples were sequenced for confirmation purposes. Fig 2 shows the representative plots obtained from real-time PCR amplification of *mcr-1*, *mcr-2* and 16S rRNA. The *mcr-1* positive strains, AR-Bank #0346 and AR-Bank #0349, have typical Ct values between 8.84 and 9.04, while the *mcr* negative strains, RM10849 and RM10850, have Ct values > 23, at least 14 cycles more than *mcr-1* positive strains. The *mcr-2* synthetic gene (SG-mcr-2) was used as a positive control for *mcr-2* PCR and the Ct value obtained was around 3 (Note: *mcr-2* positive strains were not available), indicating the effectiveness of the real-time PCR for *mcr-2* detection. All strains tested for the rRNA gene have very close Ct values ranging between 4.13 and 4.87, suggesting similar quality of DNA templates from different isolates.

The results obtained from the real-time PCR showed that all 1000 STEC isolates collected during 2006 through 2014 were negative for *mcr* genes, which was also confirmed by conventional PCR, suggesting a very low probability that *mcr* genes may be currently prevalent in STEC, recovered from a produce production region in California. An ongoing survey study by the National Antimicrobial Resistance Monitoring System on animals and meat samples also indicates the rarity of *mcr*-resistance in bacteria in the US, only one resistant strain was found among over 9000 *E*. *coli* samples [33]. Additionally, a separate screening of 2,003 samples of cecal contents from slaughtered animals yielded two single strains of colistin-resistant *E*. *coli*, recovered from a pig intestinal sample [17]. These findings indicate a lower prevalence of transferable colistin resistance in the United States when compared to other countries [17]. In the present study, the absence of transferable colistin-resistance gene in the tested STEC isolates, recovered from a leafy vegetable production region, could be due to the lack of selection pressure from limited use of colistin, giving the fact that colistin has been exclusively used in food animals, but not in fresh produce field in California [34]. In countries where the use of colistin has been uncontrolled and aggressive in animal treatment, the prevalence rate of *mcr* genes was significantly higher and the antimicrobial genes have been observed to spread across species [28, 30, 35]. It is worth to note that occurrence of new *mcr* variants could happen in the future, the conclusion made in this study is based on the PCR results for *mcr-1* and *mcr-2* only.
